# *Fastbreak*: a tool for analysis and visualization of structural variations in genomic data

**DOI:** 10.1186/1687-4153-2012-15

**Published:** 2012-10-09

**Authors:** Ryan Bressler, Jake Lin, Andrea Eakin, Thomas Robinson, Richard Kreisberg, Hector Rovira, Theo Knijnenburg, John Boyle, Ilya Shmulevich

**Affiliations:** 1 Institute for System Biology, 401 Terry Avenue North, Seattle, WA, 98109-5234, USA; 2 Division of Molecular Carcinogenesis, Netherlands Cancer Institute, Plesmanlaan 121, Amsterdam, 1066CX, The Netherlands

**Keywords:** Cancer genomics, Structural variation, Translocation

## Abstract

Genomic studies are now being undertaken on thousands of samples requiring new computational tools that can rapidly analyze data to identify clinically important features. Inferring structural variations in cancer genomes from mate-paired reads is a combinatorially difficult problem. We introduce *Fastbreak*, a fast and scalable toolkit that enables the analysis and visualization of large amounts of data from projects such as The Cancer Genome Atlas.

## Introduction

Genomic analysis of cancer and other genetic diseases is changing from the study of individuals to the study of large populations. This is exemplified by large scale projects such as The Cancer Genome Atlas (TCGA), a multi-institution consortium working to build a comprehensive compendium of genomic information that promises to reveal the molecular basis of cancer, and lead to new discoveries and therapies. Currently, TCGA centers are targeted to undertake the integrated analysis of 20-25 cancer types using more than twenty thousand samples [1,2]. This endeavor provides investigators with an unprecedented view of the genomic aberrations that define many human cancers [3]. Cancer cells display diverse genetic structure even within a single individual [4]. Analysis of these structural variations (SVs) across thousands of individuals requires tools that must execute quickly and minimize systematic bias and errors.

Structural variants can be inferred from mapped mate-pair sequencing data by analyzing read pairs that have unlikely positions or orientations relative to each other and several methods and applications for this purpose have been presented [5,6]. However, identifying groups of unlikely reads that support a particular structural variation can involve computations that become combinatorially complex as the number of reads increases. Algorithms such as BreakDancer [6] that make pairwise comparisons between reads have running times that scale nonlinearly with input size and are thus expensive to apply to large data sets consisting of many high coverage genomes. We present *Fastbreak*, a toolset that has been designed to enable efficient and parallelizable SV analysis of next-generation sequencing data. The algorithm and associated tools are available as open source software at http://code.google.com/p/fastbreak/ and incorporates several features:

**Scalable rule-based approach**: The system uses a set of rules designed to detect the signatures of SVs in a single pass over the data and accumulate this information in efficient, parallelizable data structures. These rules can be further tailored to focus on the signature of cancer-associated SVs, greatly reducing false positives (see Rules used in sample analysis).

**Robust analysis**: Because of variations in coverage and quality in the large amounts of data available, the software chains together different tools and statistical methods to identify both statistically anomalous files and those sections of the data that are free from systematic bias (see Robustness of analysis and quality assurance of data).

**Visual data mining**: The tool incorporates a set of novel visualizations allowing for interactive exploration and the presentation of the results at different scales (see Interaction visual representation).

**Figure 1:**
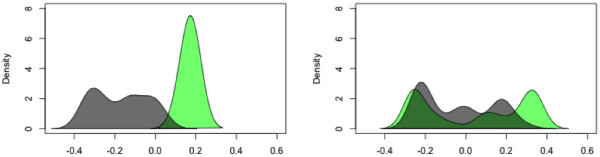
**Separation of tumor from blood by *Fastbreak *(left) and BreakDancer (right).** The separation of the tissue type (cancer versus blood), showing kernel density estimates along the first coordinate of a multidimensional scaling (MDS) solution derived from the mutual information distance [7] between samples, demonstrates that *Fastbreak* is robust against sample collection and instrumentation biases. The blood sample densities are shown in green, while the pooled cancer samples are shown in grey. For *Fastbreak* (left) the blood samples show a high c-index [8] for the blood cluster (0.97) and no significant correlation (−0.01) between SVs detected in genes and their corresponding coverage; for BreakDancer [6], the blood samples show a lower c-index for the blood cluster (0.68) and a weak correlation (0.28) between SVs detected in genes and their corresponding coverage, an undesired confounding property. Both applications were run only on samples and regions that passed our QA process. Without this restriction, variation along the primary coordinate of MDS is dominated by batch effects unrelated to tissue type.

**Figure 2:**
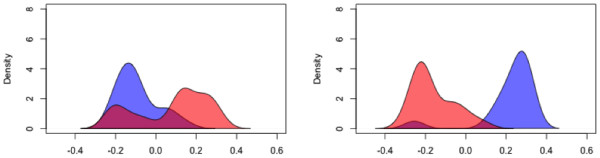
**Kernel density estimates of cancer type samples (glioblastoma versus ovarian cancer) along the first coordinate of a multidimensional scaling solution derived from the mutual information distance between samples as analyzed by *Fastbreak *(left) and BreakDancer (right).** The GBM cancer patient distribution is shown in red (with a c-index of 0.8 for *Fastbreak* and 0.94 for BreakDancer [8]), and the ovarian distribution (with a c-index of 0.7 for *Fastbreak* and 0.95 BreakDancer) in blue. The results show that *Fastbreak* can distinguish cancer types without exhibiting strong batch effects. Some of BreakDancer’s separation of samples can be attributed to batch effects, as shown in Figure 1, due to differential coverage between the two cancers.

By incorporating these features, *Fastbreak* has been used for detecting SVs in hundreds of TCGA samples and found to execute quickly and produce conservative results (Figures 1 and 2).

*Fastbreak*'s rule based approach both allows the running time to scale linearly with input size and is linearizable in the sense that the analysis of a single file can be distributed across a number of commodity servers enabling rapid analysis of large datasets. Analysis of a single sample can take hours on a single machine. The analysis of a large data set can be distributed by sample, producing linear speedups. In our own testing we were able to efficiently utilize approximately 80 cores in this manner, allowing us to process hundreds of files in days. Beyond this point, we found that the analysis was bottlenecked by the speed of our file server. If more machines are available, the linearizable nature of the analysis allows it to be further distributed using Google’s MapReduce paradigm (as implemented by Apache Hadoop) providing further log-linear speedups and eliminating the bottleneck of a single file server. Running times for various files and a comparison to BreakDancer are provided in Table 1.

**Table 1:** Running times in minutes for fastbreak, fastbreak on hadoop on a 9 server cluster, and BreakDancer

**Bam file**	**Fastbreak (both passes)**	**Fastbreak on hadoop (pass1 + pass2)**	**BreakDancer**
9 gb Tumor	80	4 + 25	785
20 gb Tumor	91	8 + 40	812
40 gb Blood	163	9 + 110	449

Hadoop running times are dominated by the time it takes the longest reducer to finish, meaning most of the cluster is unused for most of the time allowing greater throughput when processing many files. BreakDancer running times appear to scale with the number of abnormal reads, not the file size; it performs faster on the larger “blood” files than it does on the smaller “tumor” files.

The linear scaling and linearizability of the *Fastbreak* algorithm are both due to the use of efficient spatial data structures to accumulate counts of the read pairs that satisfy a set of rules in a single pass over the data. A second set of rules is then applied to all of the regions in the spatial data structure to calculate the confidence that structural variation has affected that region. The data structures are implemented for accumulating both one dimensional (the position of a single read) and two dimensional (such as the positions of two paired reads) genetic data in coarse (1000 bp) bins. The first set of rules describes what may be considered an abnormal read pair and the data structure accumulates both the density of normal and abnormal read pairs in one and two dimensions. The second set of rules identifies, classifies and scores possible structural variations based on the size of abnormal read pair clusters and the local coverage as represented in these densities. The rules are described in detail in Rules used in sample analysis.

**Figure 3:**
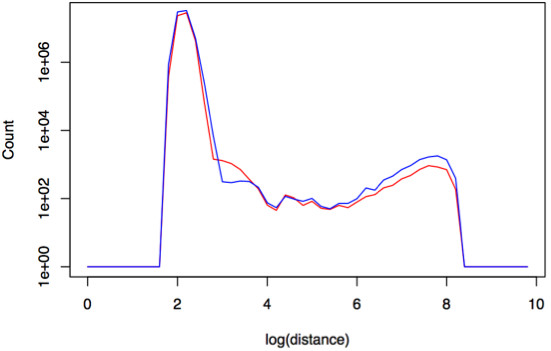
**Example output from *Fastbreak *showing a density plot of the distances between paired-end read mapped positions.** The red line represents cancer samples while blue represents the blood samples from the same patients. Across all the samples, distances between mate pairs of 1000 and 7000 base pairs were found to be more highly prevalent in tumor samples than in corresponding blood samples.

**Figure 4:**
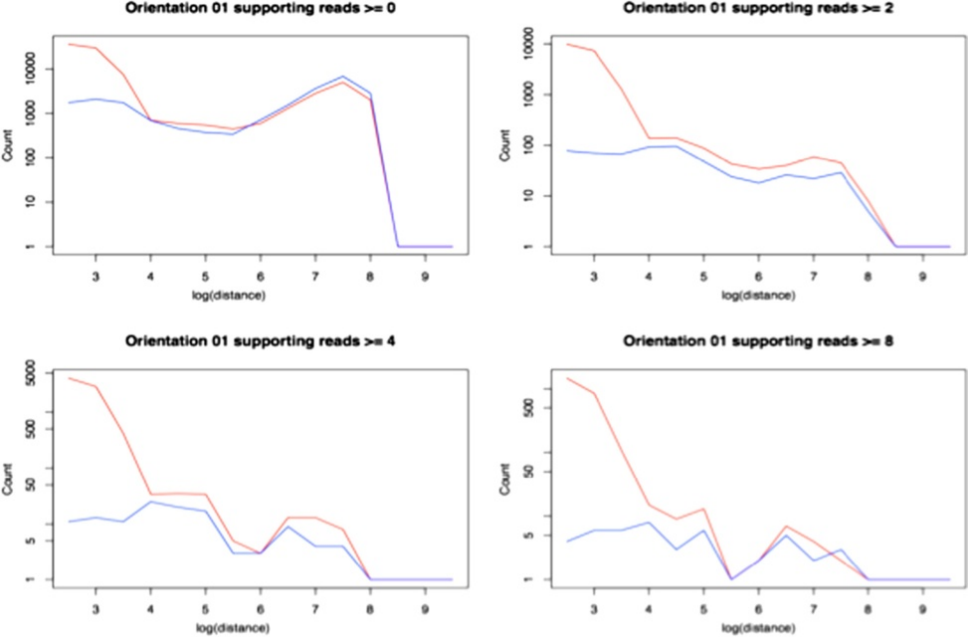
**Distance distributions of clusters of read pairs of varying sizes.***Fastbreak* also generates statistics that can be used to determine thresholds for the minimum number of supported reads required to identify features. The plots above show how larger clusters of abnormal read pairs are significantly more prevalent in tumor samples. This information is used to define the rule that a minimum of two supporting reads is required to identify abnormal behavior.

Prior to analysis, a QA procedure is applied, which also produces a nonparametric estimate of the distribution of read pair distances (Figure 3) that can be used to fine-tune the rule system. Common problems identified by this process involve issues of erroneous read groups within samples and coverage depth discrepancies (see Robustness of analysis and quality assurance of data) due to changes in protocol and platforms (e.g., during the early stages of TCGA). The rule-based system can then be optimized by executing a first pass analysis to identify which parameters give reasonable differences between paired normal/cancer samples or across other sets of related samples (see Robustness of analysis and quality assurance of data). To remove biases due to coverage differences across a large sample set, a biclustering algorithm [9] is used to select subgroups of genes/patients for direct comparison. An analysis of the genes disrupted across hundreds of ovarian cancer and glioblastoma samples (Figure 4) shows that the *Fastbreak* results can be used to distinguish between tumor and blood samples and, to a lesser extent, disease types and to identify strong similarities in the types of gene function and pathways that are disrupted by structural variation (Robustness of analysis and quality assurance of data).

**Figure 5:**
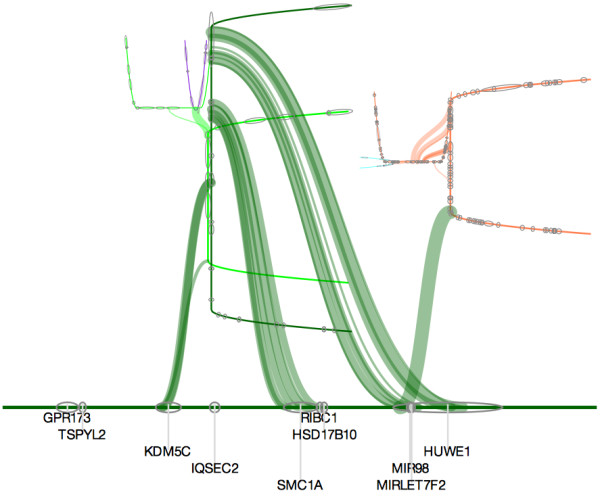
Screen shot of interactive visualization of the local genetic topology of a gene in a single sample highlights links between regions and chromosomes.

**Figure 6:**
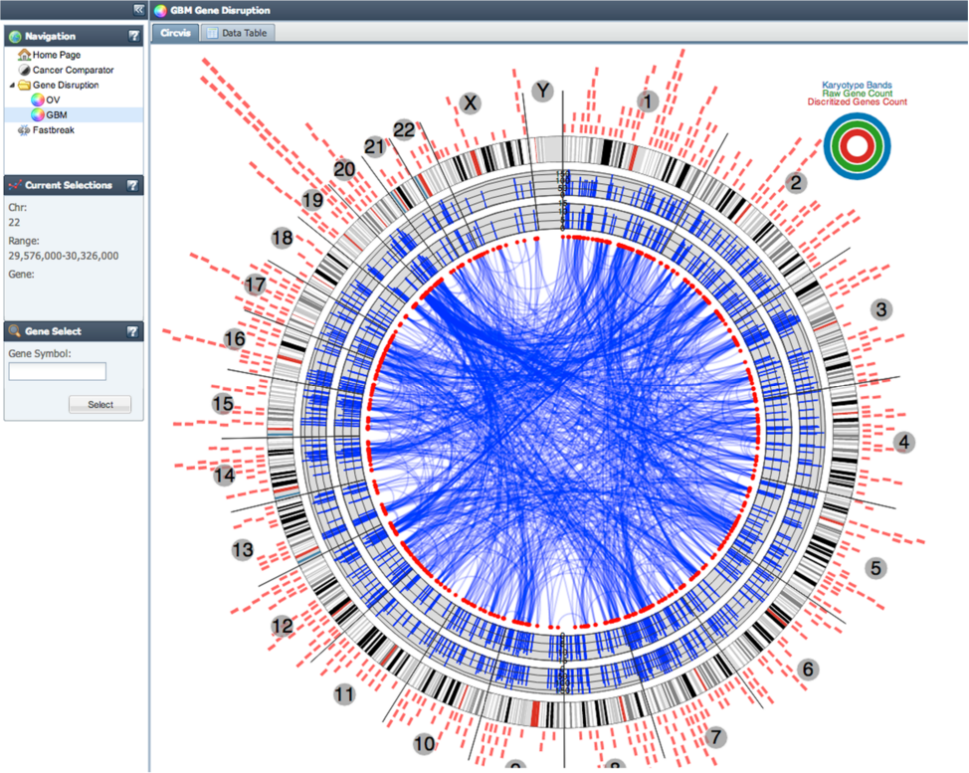
**GBM plot highlights genes with informative occurrences of structural variation across the entire cancer as measured by mutual information.** The plot is rendered using Visquick [10] which allows interactive exploration of the data.

Because it is difficult to represent such a large data set statically, we developed a dynamic web application that visualizes the results of *Fastbreak* at different scales, using a set of custom visualization components. This allows researchers to explore the effects of structural variation on a genetic level across the entire data set. The local genetic topology of a disrupted gene is a hierarchical structure of contiguous regions that may be visualized as a tree branching to different regions and chromosomes (Figure 5). The similarity (as measured by mutual information) of SVs between genes within a disease defines a network that can be visualized using an interactive circular plot (Figure 6). Comparisons between the most frequently disrupted genes in different diseases can be explored dynamically using an interactive parallel coordinates plot (Figure 5).

## Rules used in sample analysis

The *Fastbreak* system uses sets of rules, designed to detect genetic structural variations in high throughput sequence data. For analysis of glioblastoma (GBM) and ovarian cancer, these rules have been further refined to detect features that occur prevalently in disease (cancer) samples. To detect these structural variations, rules have been developed to identify three different sorts of abnormal read pairs: those with an abnormal distance between mapped positions; those with inconsistent orientation; and those mapped to different chromosomes. The algorithm compiles a list of such abnormal ("odd") reads in a linear time pass over the data. These reads are stored in a spatial data structure allowing us to identify groups of similar odd read pairs that meet our criteria in a second linear time pass over the filtered data. This data structure uses a system of bins that limits the resolution of the data, but provides significant speed advantages.

In mate pair sequence data, the resolution at which breakpoints can be confidently detected is dictated by the longest distance between mate-paired reads that can be considered normal. In the non-disease (blood) samples that were analyzed, only 0.1% of the reads had mapped distances of more than 1000 base pairs. *Fastbreak* uses this length to define the size of the bins in its internal data structure so that most normal read pairs will fall within a single bin. This eliminates the combinatorial difficulty of identifying clusters of abnormal read pairs and is one of the key optimizations that allow the algorithm’s running time to scale linearly with data size.

The rule set used in the analysis of the cancer samples was developed to identify clusters of abnormal read pairs that appear as part of a signature present in the majority of tumor samples within a set of matched (from the same patient) tumor and blood samples. *Fastbreak* was used to identify the common signature of abnormal read pairs found in tumor samples. The analysis to determine the best rule set is run separately for each disease, and across all the tumors there was found to be an enrichment of mate-pair distances between 1000-7000 bp (see Figure 3). Pair distances greater than 7000 bp are not significantly enriched in tumor samples, indicating that they are caused primarily by random noise or by structural variations present in the normal tissue relative to the reference genome used for mapping. This upper limit of this window is on the same order as the fall off of structural variations longer than 2000 bp observed by Clark *et al*. Through the deep sequencing of a GBM cell line [11].

For the comparative cancer analysis, the rule system was designed to detect structural variations supported by orientation chromosome or pair distance in this 1000-7000 bp window. Because some of these reads will be the product of random noise, an additional analysis is done to determine how many reads and what percent of the total coverage of a region are needed to conservatively identify a structural variation. These rules can be shown to maximize the difference between the distance distributions of tumor and blood samples (Figure 4).

To account for differences in mapping quality of reads, each inferred feature is assigned a score which aggregates the mapping quality assigned to all supporting reads using a probabilistic interpretation, so that the score assigned to the feature is the probability that not all of the reads were mismapped. This provides a score for each identified feature that increases with both the number of supporting reads and their mapping quality. For the analysis presented, we specified that, for us to consider a cluster of abnormal reads a structural variation, the number of reads that show unusual characteristics must be greater than two, and must account for more than 5% of the local coverage. We found that these rules are well suited to the exome sequenced samples that we analyzed, but more or less conservative rules can be used depending on the quality and coverage of the data available.

## Robustness of analysis and quality assurance of data

*Fastbreak* helps to formulate rules for the identification of biologically relevant features that are robust against false positives due to differences in coverage and other batch effects. In addition to sequencing errors, automated analyses need to remove coverage bias and sample anomalies. To minimize the effects of disparate coverage levels between samples and genes, a biclustering method has been integrated to identify a subgroup of genes and samples with relatively consistent coverage. Erroneous individual samples are removed by use of an internal QA process that analyses different read groups within a sample to find anomalies. Matched pairs that pass the QA tests are then processed for secondary analysis.

The example analysis here involves the identification of structural variants across different cancer types. The analysis used a data set of 172 GBM patients and 132 ovarian cancer patients. Of these, fewer than 50% (96 GBM samples and 38 ovarian samples) passed the QA test process (see below). The parameters of the tests can be changed to include more patients, either through analysis of fewer chromosome regions, or by lowering the quality/coverage thresholds.

The QA process is designed to identify biases across and within samples, and identify chromosome regions across patients that can be compared. The system identifies regions that have sufficient coverage across patients, so that biases due to coverage depth are minimized. Batch effects can be studied by looking for correlations between coverage and identified features (a generally undesired property), and by comparing across samples (see Figures 1 and 2). As *Fastbreak* can be optimized to identify features using rules specific to the system under study, and can compensate for differences in coverage, it shows some robustness to changes in conditions and corresponding batch effects.

The functional significance of the analysis is suggested by the enrichment of genes related to functions such as extracellular matrix and focal adhesion in the list of most disrupted genes (see Tables 2 and 3). Complete details of the samples used and a complete list of disrupted genes are given on the accompanying web site. The functional significance of the disrupted genes identified is shown in Table 3. The gene disruptions can be mined using the interactive web application outlined in Interaction visual representation (http://fastbreak.systemsbiology.net). All code associated with the web application, and analysis systems, is made freely available under an open source public license.

**Table 2:** Most disrupted genes across the ovarian cancer and glioblastoma cancer data sets

**Gene name**	**Number of disrupted GBM samples**	**Gene name**	**Number of disrupted ovarian cancer samples**
DNAH9	43	KALRN	17
SYNE1	42	MTOR	17
SYNE2	40	TG	16
TG	40	PAPPA2	15
KALRN	38	SYNE1	15
TRRAP	38	TRIO	15
MLL3	37	CACNA2D3	14
PKHD1	27	TECTA	14
RELN	37	ANK1	13
DNAH8	36	CIT	13

**Table 3:** Functional enrichment of most structurally disrupted genes in pooled GBM and ovarian cancer samples

**Functional group**	**Enrichment**
Extracellular matrix	7e-6
Focal Adhesion	2e-5
Phospoprotein	3e-5
Guanyl-nucelotide exchange factor	5e-4
Cell morphogensis	7e-4
Axonogenesis	1e-3

The analysis was performed using the NIH DAVID tool [12,13] (March 2011), with the background population being those identified through the biclustering step of the *Fastbreak* analysis. The p-values are multi-test corrected using the default NIH David method (Benjamini–Hochberg correction).

## Interactive visual representation

**Figure 7:**
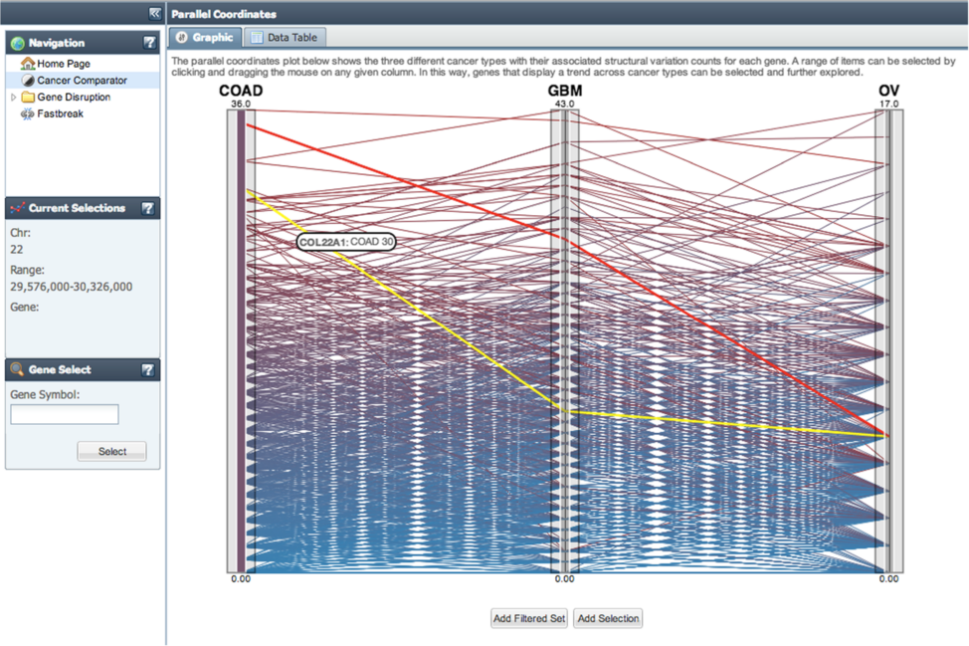
Cancer Comparator Interactive Parallel Coordinates Plot allows the user to explore the relative rank of genes with structural variations across colon adenocarcinoma (COAD), glioblastoma multiforme (GBM) and ovarian cancer (OV).

A web application providing visualizations of *Fastbreak* analysis results was developed to enable an end-user to explore the data in an intuitive manner. The application allows a user to view data at three different levels, as well as search specific regions by chromosome coordinates or gene names. To explore similarities or differences between cancers, genes that have high structural variation are visualized in a parallel coordinates plot on the cancer comparator tab (see Figure 7). Mouse-over events and an alternate table view allow the user to view specific information regarding points on the plot and number of disruptions found for a particular gene.

Gene behaviors within a cancer type can be explored by selecting the OV (ovarian) or GBM (glioblastoma) tab on the left side of the application (see Figure 6). The cancer specific visualization level shows mutual information distances between genes across all patients as a circular plot. Again, mouse-over events and alternate table views can be used to view the data in more detail.

The third level of visualization allows a user to view structural variations at a specific location for a selected patient sample. Comparisons between tissues (tumor and blood) and patients can be done at this level of the application. Selection of patients and chromosome location can be done in the “data and range selection” window, while selection of parameters specific to the visualization can be altered in the “advanced parameters” window. A depth-first graph traversal of the structural variant data is used as the underlying data of the visualization. Results are drawn as a cyclic tree such that each contiguous region is represented by a pair of orthogonal branches. Gene location is shown along the base and branches of the trees while coverage information is displayed below the tree. The thickness of the branches indicates the number of supporting reads for the particular structural variation event. Mouse-over and click events are also implemented to view more information regarding a specific SV event. The organic structure of this visualization allows the viewer to quickly distinguish between different topologies based on qualitative differences in tree appearances (Figure 5).

The web application described above may be viewed and explored at http://fastbreak.systemsbiology.net. A download of the underlying data and web application are also available on the site.

## Conclusions

**Figure 8:**
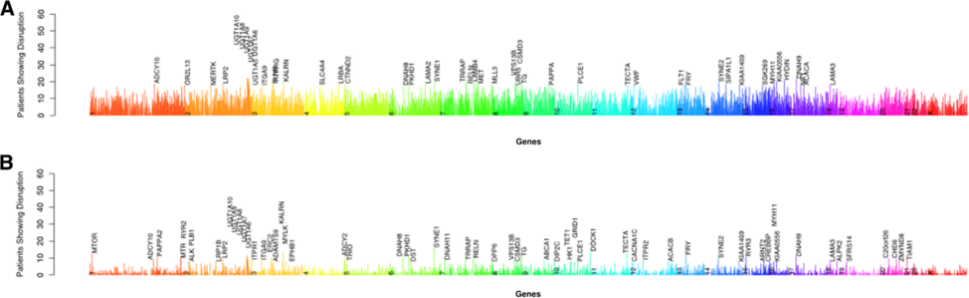
**The genes that are most commonly disrupted in glioblastoma (A) and ovarian cancer (B).** Genes are arranged in order by chromosome and position and only well covered genes are indicated.

The approach implemented has several advantages over existing approaches. *Fastbreak*’s rule-based algorithm can be used to reliably and conservatively identify structural variants of biological significance in the TCGA data set. In terms of resistance to bias correlated with the diverse levels of coverage seen in the exome data, our results improve upon those produced by BreakDancer [5], which was not designed with exome data in mind (see Figures 1 and 2). We have further shown that this approach can be easily parallelized across commodity servers, allowing the rapid analysis of petabyte-scale data sets and provided a new tool for dynamically visualizing and exploring the genetic topology of cancer samples inferred by *Fastbreak*. The combination of these approaches allows one to produce a novel population-scale view of genetic structural variation within and across cancers (Figure 8).

However, *Fastbreak* provides only a coarse view of structural variation. It can be used to identify the regions that have been affected by structural variation, but does not attempt to describe precisely what variation has occurred. It is our hope that future tools might use *Fastbreak*-like data structures and approaches to parallelization to accelerate more precise algorithms.

## Competing interests

The author(s) declare that they have no competing interests.
